# Case report: Autosomal recessive type 3 Stickler syndrome caused by compound heterozygous mutations in *COL11A2*

**DOI:** 10.3389/fgene.2023.1154087

**Published:** 2023-06-06

**Authors:** Ying Su, Chun-Qiong Ran, Zhe-Long Liu, Yan Yang, Gang Yuan, Shu-Hong Hu, Xue-Feng Yu, Wen-Tao He

**Affiliations:** ^1^ Division of Endocrinology, Department of Internal Medicine, Tongji Hospital, Tongji Medical College, Huazhong University of Science and Technology, Wuhan, China; ^2^ Branch of National Clinical Research Center for Metabolic Diseases, Wuhan, China

**Keywords:** Col11a2, splicing mutation, case report, type 3 Stickler syndrome, genetic counseling

## Abstract

**Background:** Stickler syndrome (SS) is a group of hereditary collagenopathies caused by a variety of collagen and non-collagen genes. Affected patients have characteristic manifestations involving ophthalmic, articular, craniofacial and auditory disorders. SS is classified into several subtypes according to clinical and molecular features. Type 3 SS is an ultra-rare disease, known as non-ocular SS or otospondylomegaepiphyseal dysplasia (OSMED) with only a few pathogenic *COL11A2* variants reported to date.

**Case presentation:** A 29-year-old Chinese male was referred to our hospital for hearing loss and multiple joint pain. He presented a phenotype highly suggestive of OSMED, including progressive sensorineural deafness, spondyloepiphyseal dysplasia with large epiphyses, platyspondyly, degenerative osteoarthritis, and sunken nasal bridge. We detected compound heterozygous mutations in *COL11A2*, both of which were predicted to be splicing mutations. One is synonymous mutation c.3774C>T (p.Gly1258Gly) supposed to be a splice site mutation, the other is a novel intron mutation c.4750 + 5 G>A, which is a highly conservative site across several species. We also present a review of the current known pathogenic mutation spectrum of *COL11A2* in patients with type 3 SS.

**Conclusion:** Both synonymous extonic and intronic variants are easily overlooked by whole-exome sequencing. For patients with clinical manifestations suspected of SS syndrome, next-generation whole-genome sequencing is necessary for precision diagnosis and genetic counseling.

## Introduction

Stickler syndrome (SS) is a group of multisystem collagenopathies characterized by ophthalmic, auditory, craniofacial, articular and skeletal abnormalities. The first case of “hereditary progressive arthro-ophthalmopathy” was reported by Stickler *et al* dates back to 1965 (Stickler et al., 1965). SS is clinically and molecularly heterogeneous, which makes recognition and precise diagnosis of the disease difficult. Currently, pathogenic variants in six collagen-type genes (*COL2A1*, *COL11A1*, *COL11A2*, *COL9A1*, *COL9A2* and *COL9A3*) and at least five non-collagen genes (*LRP2*, *GZF1*, *BMP4*, *PLOD3* and *LOXL3*) have been demonstrated to be involved in the pathogenesis of SS (Schrauwen et al., 2014; Alzahrani et al., 2015; Chan et al., 2019; Ewans et al., 2019; Nixon et al., 2019). The abnormal extracellular matrix modelling in affected tissues caused by genetic mutations leads to characteristic manifestations in patients with SS. To date, it has been delineated that more than 40 different collagen or collagen-related genes are responsible for synthesizing and assembling at least 29 types of collagens (Carter and Raggio, 2009). Nevertheless, the most causative genetic alterations in SS are associated with abnormal production or assembly of fibrillar collagens II, IX and XI, which are enriched in the vitreous humor as well as hyaline and elastic cartilage (Ihanamaki et al., 2004).

Type 1 SS (OMIM #108300), accounting for 80%–90% of cases, is featured by a high risk for blindness resulting from retinal detachment and membranous vitreous changes. The haploinsufficiency of *COL2A1*, the encoding gene for collagen type II alpha one chain, is responsible for autosomal dominant type 1 SS (Roche and Cresswell, 1991).

Type 2 SS (OMIM #604841), representing the remaining 10%–20% of cases, is usually caused by monoallelic mutations in *COL11A1,* which encodes the collagen type XI alpha one chain (Soh et al., 2022). However, biallelic pathogenic variants of *COL11A1* have also been reported occasionally (Nixon et al., 2022). Type 2 SS is characterized by a beaded vitreous phenotype, different from type 1 SS.

Type 3 SS (OMIM #184840) is a rare form, previously known as non-ocular SS, or otospondylomegaepiphyseal dysplasia (OSMED) (OMIM #184840, #215150), or Weissenbacher Zweymüller syndrome. The first detailed description of this syndrome was made by pediatricians, Dr. Weissenbacher and Zweymüller, in 1964 (Weissenbacher and Zweymueller, 1964). The affected patient was a normal-sized newborn with several signs of chondrodysplasia, including a markedly snub nose, coronal vertebral clefts, and rhizomelic shortness of limbs with dumb-bell-shaped femora (Weissenbacher and Zweymueller, 1964; Pihlajamaa et al., 1998). The patient did not have ocular signs such as myopia and vitreoretinal degeneration. The follow-up results of the original patient showed that he had developed sensorineural deafness at 5 years old, and spondyloepiphyseal dysplasia at about 13 years old (Giedion et al., 1982; Pihlajamaa et al., 1998). After summarizing the common features of the four affected patients, Giedion *et al.* had postulated the nomenclature, OSMED, to characterize this condition better (Giedion et al., 1982). The pathogenic gene responsible for OSMED was identified by directed sequencing of two candidate genes (*COL2A1* and *COL11A2*) expressed in cartilage (Pihlajamaa et al., 1998). Finally, *COL11A2* mutation has been demonstrated to be the etiology of OSMED. Both autosomal dominant and recessive inheritable patterns of *COL11A2* mutation have been documented to be involved in the pathogenesis of type 3 SS (Vikkula et al., 1995; Melkoniemi et al., 2000), depending on the mutation sites.

The uncommon causative genes for SS include coding genes for collagen IX protein, namely, *COL9A1*, *COL9A2* and *COL9A3*, together with non-collagen genes mentioned previously.

In this report, we describe a 29-year-old Chinese male with non-ocular OSMED-like manifestations. Whole-genome sequencing of DNA from the peripheral blood has uncovered a compound heterozygous mutation of *COL11A2*. We also review the currently known pathogenic mutations of *COL11A2* for type 3 SS.

## Case description

**FIGURE 1:**
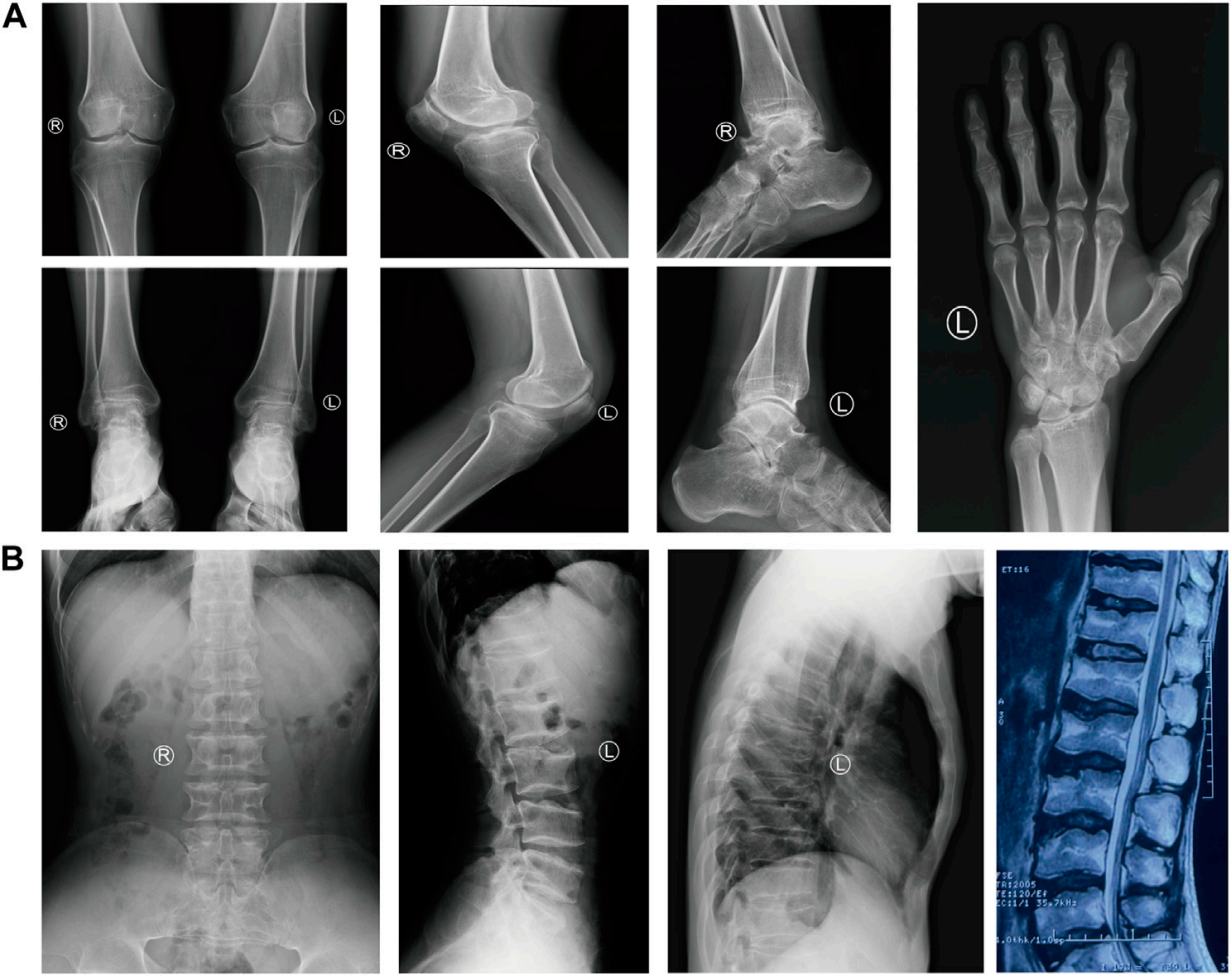
The radiological findings of osteoarthritis in the proband. **(A)** Radiographs of the knees, ankles, left hand and foot, showing the diffuse joint-space-narrowing. **(B)** Radiographs and MRI of the spine, showing platyspondyly.

**FIGURE 2:**
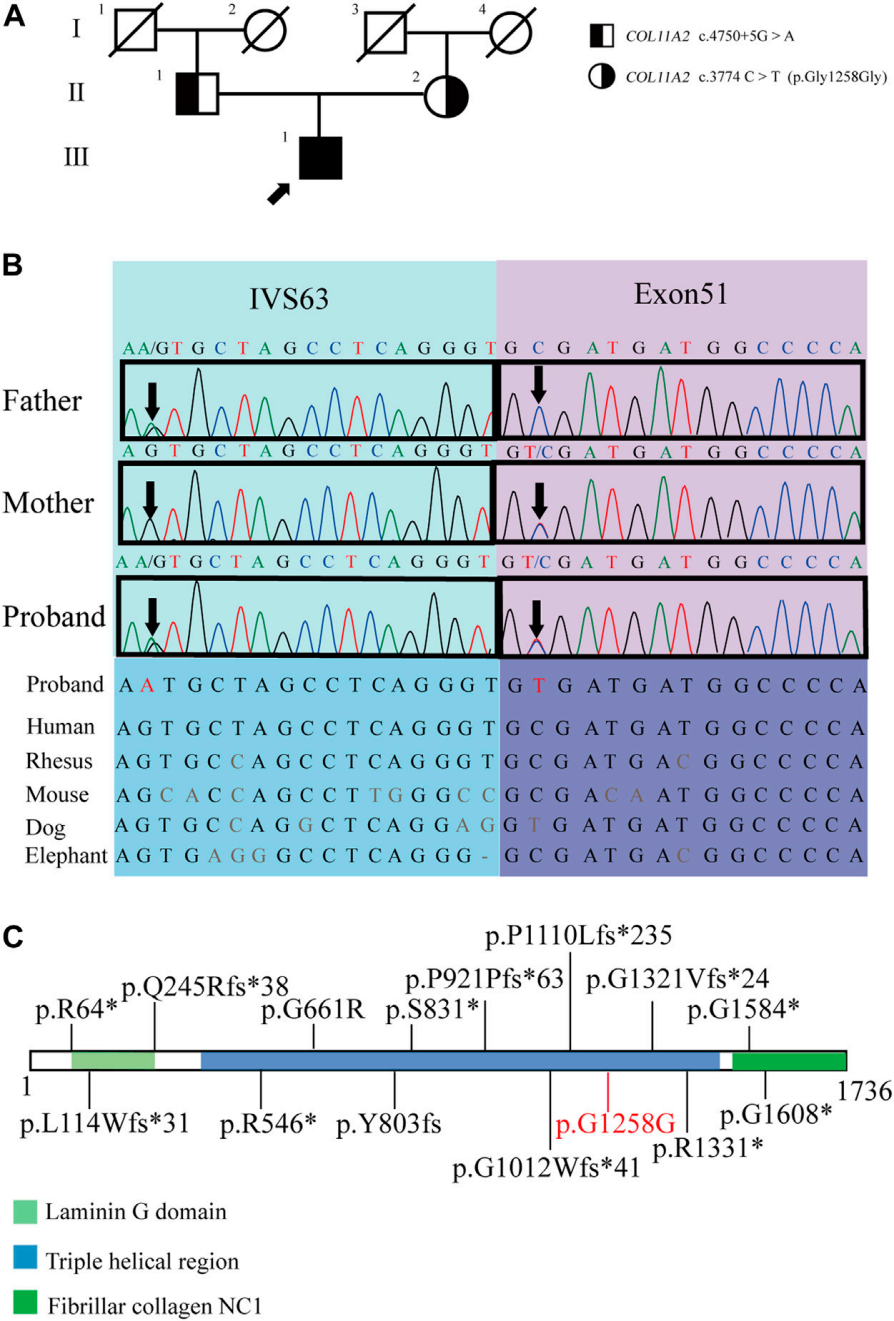
Pedigrees of the proband and the genetic characteristics of the pedigree. **(A)** Pedigrees of the proband (arrow indicates proband). **(B)** The Sanger sequencing of the family and the evolutionary conservation of the cluster across multiple species. **(C)** *COL11A2* variants associated with type 3 SS, variants were collected from ClinVar and previously reported (variants written in black), the pathogenic variant in the proband is marked in red (the suspected splicing mutation *COL11A2* c.4750+5G > A is not shown) (https://legacy.uniprot.org/uniprot/P13942).

**FIGURE 3:**
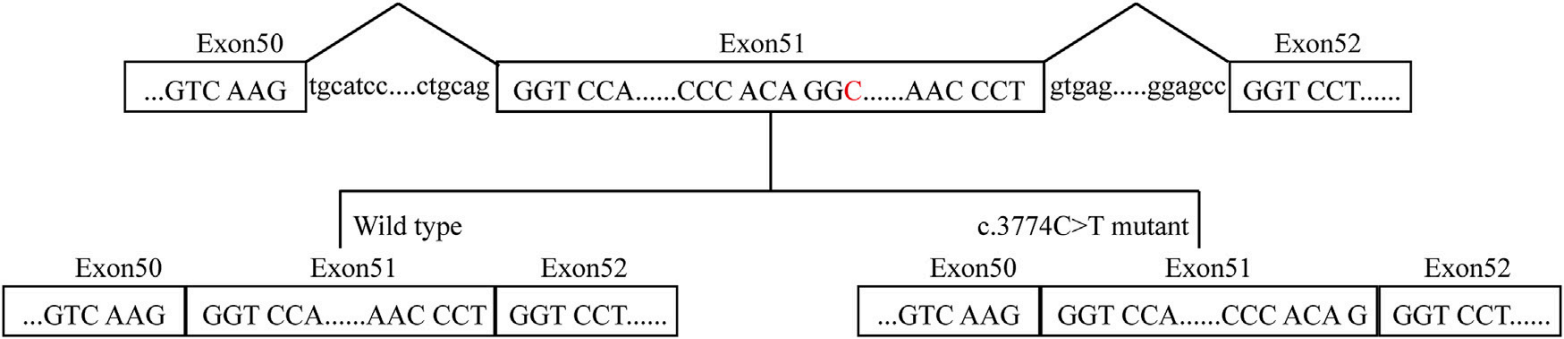
A proposed model for the role of the *COL11A2* c.3774C>T mutation. The red C represents the mutation site.

A 29-year-old Chinese male was admitted to our hospital for recurrent pain in the low back, knee and ankle joints, which had troubled him for 2 years. He was from a non-consanguineous family. He had been born with normal weight and height. During his infancy, he was noted to have enlarged interphalangeal joints and O-shaped legs. Non-progressive hearing impairment occurred since childhood, so his language development was retarded. His mental development seemed normal. No visual signs or symptoms had been observed. No similar symptoms occurred in his family members. On admission, he was 168 cm in height and 54.7 kg in weight (body mass index: 19.3 kg/m^2^). Mild abnormal body proportions were observed, with a shorter upper part compared to the lower part (80 cm vs. 88 cm). In addition, his arm span was shorter than his height (160 cm vs. 168 cm). The patient had a snub nose, a high arched palate, micrognathia, and bilateral clinodactyly of the finger and toe joints. The ophthalmologic evaluation showed normal lens, vitreous, fundus and visual acuity. According to an audiological examination, the patient has congenital bilateral sensorineural hearing loss. His middle and inner ear space appeared unremarkable with a magnetic resonance imaging (MRI) scan. Routine laboratory examinations were all negative, including erythrocyte sedimentation rate, complete blood cell count, antinuclear antibody, rheumatoid factor, thyroid hormone profiles and glucose metabolism. Body mass density (BMD) of the lumber spine and femur showed normal BMD and osteoporosis, respectively (Z-score: L1-4: −0.3, −0.5, 0, −0.2; femur: −2.5). The procollagen type 1 N-terminal prope-peptide (P1NP) was 83.2 ng/mL (9.06–76.24 ng/mL), and the carboxy-terminal cross-linked telopeptide of type 1 collagen (CTX) was 0.54 ng/mL (0.043–0.783 ng/mL). A radiographic examination revealed moderate to severe osteoarthritis in his metacarpophalangeal joints, wrists, knees and ankles. He also had various degrees of flattening of the vertebral bodies due to compression fracture (Figures 1A, B). Given the involvement of systematic chondroskeleton in the proband, we advised the performance of genetic diagnosis. After the consent of the patient and his family members, we employed high-throughput sequencing of the whole-genome of the proband’s peripheral blood. It revealed that the proband harbored compound heterozygous mutations in *COL11A2*. One is c.3774 C > T and the other is c.4750 + 5G > A (Figures 2A, B). The two mutations were confirmed by Sanger’s sequencing both in the proband and his parents. *COL11A2* c.3774 C > T is located in a consensus sequence of 5′ splice site (Figure 3). Base on the American College of Medical Genetics and Genomics (ACMG) guidelines, the variation is considered to be uncertain significance. Therefore, we investigated the influence of this synonymous mutation on splicing process *in silico* (NetGene2, NNSPLICE0.9) (Table 1). These analyses suggested that c.3774 C > T may result in the aberrant 5′ splice site. He was treated with bisphosphonates, calcium and Vitamin D to alleviate osteoporosis. The pains in multiple joints were treated with nonsteroidal anti-inflammatory drugs or gabapentin intermittently. Primers used to amplify the exons of *COL11A2* were shown in Supplementary Table S1.

**TABLE 1:** *In silico* splicing simulation of *COL11A2* c.3774 C > T.

	Authentic 5’ splice site			Aberrant 5’ splice site	
	WT	Mutant		WT	Mutant
NetGene2	0	n.d		n.d	0.7
NNSPLICE0.9	0.94	n.d		0.94	0.78

WT, wild type; n.d, not detected. A score ranges between 0 and 1 for potential splice site, The higher the score, the higher the probability of variable splicing (Jian et al., 2014).

## Discussion

In this study, the patient was diagnosed with autosomal recessive type 3 SS based on clinical manifestations, imaging findings and genetic sequencing. As an uncommon cause of SS, type 3 SS is also known as non-ocular SS or OSMED. For the patient, one of the compound heterozygous mutations has been discovered in similar patients (c.3774C>T (p.Gly1258Gly) (Acke et al., 2014), and the other is an intron mutation site that locates in a highly-conservative site across species. Both mutations are supposed to cause abnormal splicing mutations which affect the function of type XI collagen.

Type XI collagen is a quantitatively minor component of collagen in cartilage, which co-polymerizes with type II and type IX collagen to form a heterotrimer. Type XI collagen is mainly composed of three types of chains, namely, α1 (XI), α2 (XI), and α3 (XI). *COL11A2* is the coding gene for the α2 (XI) chain (Bruckner and van der Rest, 1994). Hence, defects in *COL11A2* will only affect osteoarthritis with the normal ocular phenotype (Snead et al., 2011).

The mutation sites of the patient are both located in the triple helix region of collagen (Figure 2C), which is the sequence repetition of the amino acid triad -Gly-X-Y-. The majority of -X-Y- consists of prolines and hydroxyprolines that are essential for the formation and stabilization of the triple helix. The mutation sites in this region may lead to changes in the structure of the triple helix and corresponding functional changes (Snead et al., 2011; Chakchouk et al., 2015).

*COL11A2* gene mutations cause different clinical manifestations depending on mutation types. Missense mutations in the helix domain may destroy the stability of the helix structure. When mutations occur at the carboxyl end, namely, the starting point of the collagen helical trimer, it will result in the most serious clinical phenotype (Chen et al., 2005). It has long been recognized that synomymous mutation has no functional impact. However, growing studies have shown that synonymous mutations affect DNA, RNA, and protein-based features (Vuoristo et al., 2004; Hunt et al., 2014; Zeng et al., 2021). The *COL11A2* c.3774 C > T is close to the marginal zone of exons and may cause splicing abnormalities. The other mutation site, c.4750 + 5G>A, is an intron mutation that has not previously been reported. According to the ACMG guidelines, the variation is considered uncertain significance. Currently, it cannot be ruled out that deep intron mutations could result in disease (Snead et al., 2022). Therefore, we investigated the influence of c.3774 C > T on the *COL11A2* mRNA splicing process *in silico* (Table 1), which resulted in aberrant splicing via the aberrant 5′ splice site (Figure 3). The proband’s parents both are asymptomatic with a heterozygous mutation.

Based on the characteristics of the vitreous, a taxonomy of the SS subgroups has developed over the past 20 years. It has been shown that this taxonomy is a reliable strategy for both diagnosing SS and directing molecular study (Nixon et al., 2022; Soh et al., 2022). Type 3 SS is a non-ocular type of SS, manifesting as prelingual development of sensorineural hearing loss, growth of the epiphyses with generalized shortening of the limbs, and vertebral body dysplasia. Distinguishing facial characteristics include an upturned nose, a low nasal bridge, and midface hypoplasia. The condition appears radiographically to impact numerous joints. The upper and lower extremity’s long bones are frequently seen to be shorter, with extensive epiphyses and metaphyseal flare that give them a dumbbell form. The coronal clefts in the spine’s vertebral bodies indicate faulty ossification, making the vertebral bodies platyspondyly. Despite having shorter stature as children, most patients grow to be average height. Patients typically experience joint discomfort due to prevalent arthritic changes. Clinically, the possibility of type 3 SS should be considered in patients with early sensorineural deafness, skeletal abnormalities, and imaging alterations. Genetic detection should be carried out as soon as possible to determine the cause.

At present, there is no efficient treatment for SS. Individualized treatment seems appropriate according to the different symptoms and signs. Early-stage surgical repair of skeletal abnormalities in children with type 3 SS has been reported in the past, and the short-term outcome is favorable. However, as age increases and the disease symptoms worsen, the surgical intervention becomes more challenging (Williams et al., 2020).

There are several limitations to our case report. First of all, we did not perform any additional functional testing. It still needs to be established whether the synonymous mutation in this patient leads to alternative splicing. Secondly, due to the paucity of clinical awareness of the disease, the patient had spent a lot of time on disease diagnosis. SS should be considered when patients exhibit typical deafness, bone abnormalities, and other features. In the end, although the patient has type 3 SS, there is currently no effective treatment. However, the validation of the causative gene is beneficial for genetic counseling.

In conclusion, we report a rare case with autosomal recessive type 3 SS. Both intronic and synonymous mutations are easily missed by conventional whole-exome sequencing. Next-generation whole-genome sequencing, *in silico* prediction, and possibly supplementary functional analysis are warranted for the accurate diagnosis of these patients.

## Data Availability

The datasets for this article are not publicly available due to concerns regarding participant/patient anonymity. Requests to access the datasets should be directed to the corresponding author.

## Abbreviations

SS, Stickler syndrome; COL11A2, collagen alpha-2 (XI) chain; OSMED, otospondylomegaepiphyseal dysplasia; COL2A1, collagen alpha-1(II) chain; COL11A1, collagen alpha-1 (XI) chain; COL9A1, collagen alpha-1(IX) chain; COL9A2, collagen alpha-2(IX) chain; COL9A3, collagen alpha-3(IX); LRP2, low-density lipoprotein receptor-related protein 2; GZF1, GDNF-inducible zinc finger protein 1; BMP4, bone morphogenetic protein 4; PLOD3, multifunctional procollagen lysine hydroxylase and glycosyltransferase LH3; LOXL3, lysyl oxidase homolog 3; MRI, magnetic resonance imaging; BMD, body mass density; P1NP, procollagen type 1 N-terminal prope-peptide; CTX, carboxy-terminal cross-linked telopeptide of type 1 collagen.
