# Supplementary material for: Case report: Autosomal recessive type 3 Stickler syndrome caused by compound heterozygous mutations in COL11A2
Source: Front Genet. 2023 Jun 6;14:1154087. doi: 10.3389/fgene.2023.1154087 (PMC10279880; doi:10.3389/fgene.2023.1154087)
Supplement: Supplementary file 1 [file Table1.DOCX]

Table S1. Primers used to amplify the exons of *COL11A2*

| NO. | Primer Name | Primer sequence(5' to 3') |
| --- | --- | --- |
| 1 | COL11A2_IVS63_n1F1 (396 bp) | TGTTCAGGTGCAAACAGGTGTG |
| 2 | COL11A2_IVS63_n1R1 (396 bp) | GAGATCTTTGGCTCACTCGACT |
| 3 | COL11A2_EX51_n1F1 (433 bp) | TCTCTCACTTTCTCTCCGGATC |
| 4 | COL11A2_EX51_n1R1 (433 bp) | ACTCCCCTTGTAGGCTTGACTT |
| Note: IVS denotes intron. EX denotes exon. Forward (F) and reverse (R) primers are shown. | | |
